# Myeloid IPMK promotes the resolution of serum transfer-induced arthritis in mice

**DOI:** 10.1080/19768354.2021.1952305

**Published:** 2021-07-12

**Authors:** Hyoungjoon Ahn, Jong Seong Roh, Seulgi Lee, Jiyoon Beon, Beomgu Lee, Dong Hyun Sohn, Seyun Kim

**Affiliations:** a Department of Biological Sciences, Korea Advanced Institute of Science and Technology (KAIST), Daejeon, Korea; b Department of Herbal Prescription, College of Korean Medicine, Daegu Haany University, Gyeongsan, Korea; c Department of Microbiology and Immunology, Pusan National University School of Medicine, Yangsan, Korea; d KAIST Institute for the BioCentury, KAIST, Daejeon, Korea

**Keywords:** Rheumatoid arthritis, inflammation, inositol polyphosphate, resolution, IPMK

## Abstract

Rheumatoid arthritis (RA) is a chronic autoimmune disease characterized by widespread joint inflammation, which leads to joint damage, disability, and mortality. Among the several types of immune cells, myeloid cells such as macrophages are critical for controlling the pathogenesis of RA. Inositol phosphates are water-soluble signaling molecules, which are synthesized by a series of enzymes including inositol phosphate kinases. Previous studies revealed actions of inositol phosphates and their metabolic enzymes in the modulation of inflammation such as Toll-like receptor-triggered innate immunity. However, the physiological roles of inositol polyphosphate (IP) metabolism in the regulation of RA remain largely uncharacterized. Therefore, our study sought to determine the role of inositol polyphosphate multikinase (IPMK), a key enzyme for IP metabolism and various cellular signaling control mechanisms, in mediating RA. Using myeloid cell-specific IPMK knockout (KO) mice, arthritis was induced via intraperitoneal K/BxN serum injection, after which disease severity was evaluated. Both wild-type and IPMK KO mice developed similar RA phenotypes; however, conditional deletion of IPMK in myeloid cells led to elevated arthritis scores during the resolution phase, suggesting that IPMK deficiency in myeloid cells impairs the resolution of inflammation. Bone marrow-derived IPMK KO macrophages exhibited no apparent defects in immunoglobulin Fc receptor (FcR) activation, osteoclast differentiation, or resolvin signaling. Taken together, our findings suggest that myeloid IPMK is a key determinant of RA resolution.

## Introduction

Rheumatoid arthritis (RA), a prevalent autoimmune disease, is characterized by bone and cartilage degeneration, synovial hyperplasia, and inflammatory cell infiltration within the joints, which lead to chronic pain, functional disability, and premature mortality (McInnes and Schett 2011; Smolen et al. 2018). Monocytes and macrophages are major inflammatory cells that synthesize and secrete proinflammatory cytokines [e.g. tumor necrosis factor-α (TNF-α), interleukin (IL)-1β, and IL-6] (Brennan and McInnes 2008; Siouti and Andreakos 2019), which are major drivers of RA pathogenesis. Although pro-inflammatory macrophages are critical for the induction phase of RA, macrophages are also key mediators of chronic resolution of inflammation throughout the clearance of apoptotic cells (i.e. efferocytosis) (Watanabe et al. 2019). Therefore, elucidating the molecular mechanisms that modulate macrophage activity is key to understanding RA development and elucidate effective therapeutic targets to prevent disease progression.

Inositol polyphosphate multikinase (IPMK) is a promiscuous kinase with broad substrate specificity that catalyzes the biosynthesis of inositol polyphosphates (e.g. inositol 1,4,5,6-tetrakisphosphate, inositol 1,3,4,5,6-pentakisphosphate) as well as phosphatidylinositol 3,4,5-triphosphates (Chakraborty et al. 2011; Lee et al. 2021). In addition to its catalytic role in determining inositol phosphate and phosphatidylinositol metabolism, the non-catalytic activities of IPMK mediate various biological processes such as growth, stress response, and energy homeostasis via direct interactions with signaling factors such as the mechanistic target of rapamycin (mTOR), AMP-activated protein kinase (AMPK), liver kinase B1 (LKB1), p53, and serum response factor (SRF) (Kim et al. 2011; Bang et al. 2012, 2014; Xu et al. 2013; Kim et al. 2013). Accumulating evidence thus suggests that IPMK is a central mammalian signaling network node (Lee et al. 2021).

Previously, we demonstrated that IPMK deletion in myeloid cells protects mice against lipopolysaccharide (LPS)-induced inflammation (Kim et al. 2017). Concretely, IPMK in macrophages mediates Toll-like receptor (TLR) signaling via the interaction between IPMK and tumor necrosis factor receptor-associated factor 6 (TRAF6), which is the key member of the TLR signaling pathway. However, although IPMK in myeloid cells appears to be a critical contributor to acute septic conditions, the involvement of IPMK in chronic inflammation under pathological RA conditions remains unsolved. It is also worth noting that genome-wide association studies (GWAS) from patients with immune-mediated diseases including RA revealed single-nucleotide polymorphisms in IPMK, suggesting IPMK as a candidate risk factor for RA (Yokoyama et al. 2016). Therefore, to characterize the role of myeloid IPMK in the control of RA, we analyzed the *in vivo* phenotypes of IPMK KO mice using a K/BxN serum transfer RA model, after which the activities of IPMK KO bone marrow-derived macrophages were compared to those of control cells.

## Results

### Deletion of myeloid IPMK aggravates inflammatory arthritis

**Figure 1.**
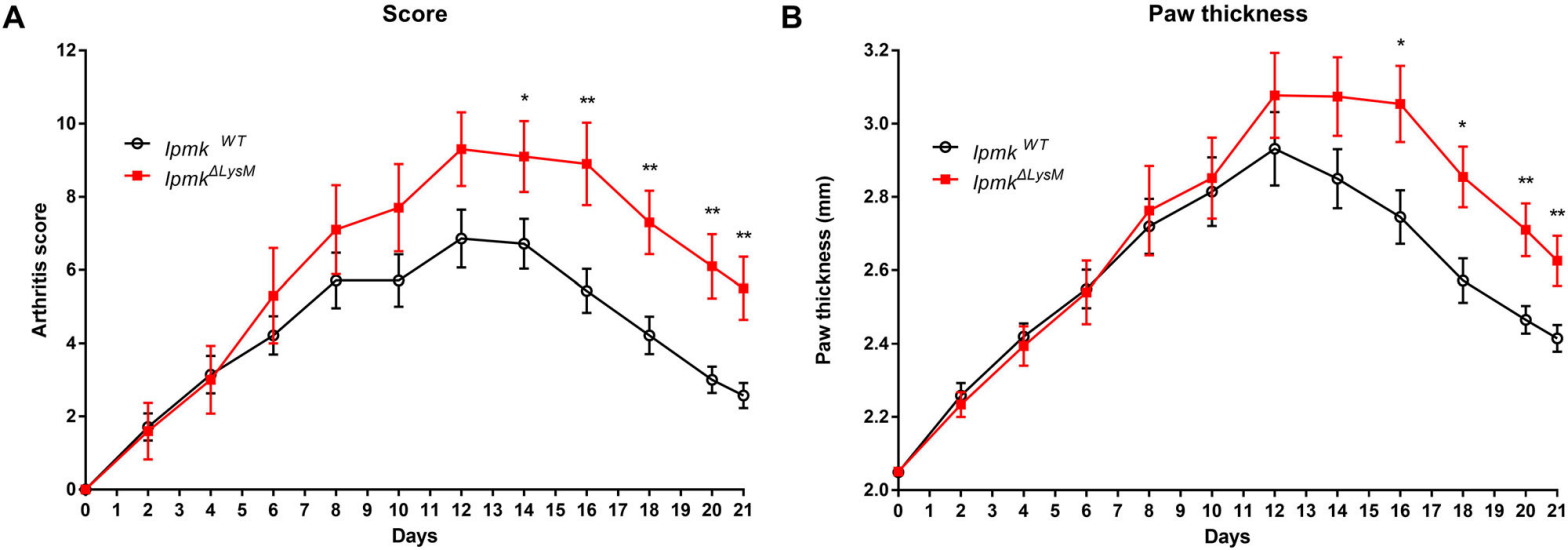
Myeloid IPMK inhibits the severity of inflammatory arthritis. (A) and (B) K/BxN serum-transfer arthritis was induced in wild-type (*Ipmk^WT)^* (*n* = 14) or myeloid-specific IPMK KO (*Ipmk^ΔLysM^*) mice (*n* = 10) by intraperitoneal injection of K/BxN serum on day 0 and 2. Arthritis score (A) and paw thickness (B) were assessed. Data are shown as mean ± standard error of the mean (SEM). *p < 0.05, **p < 0.01.

**Figure 2.**
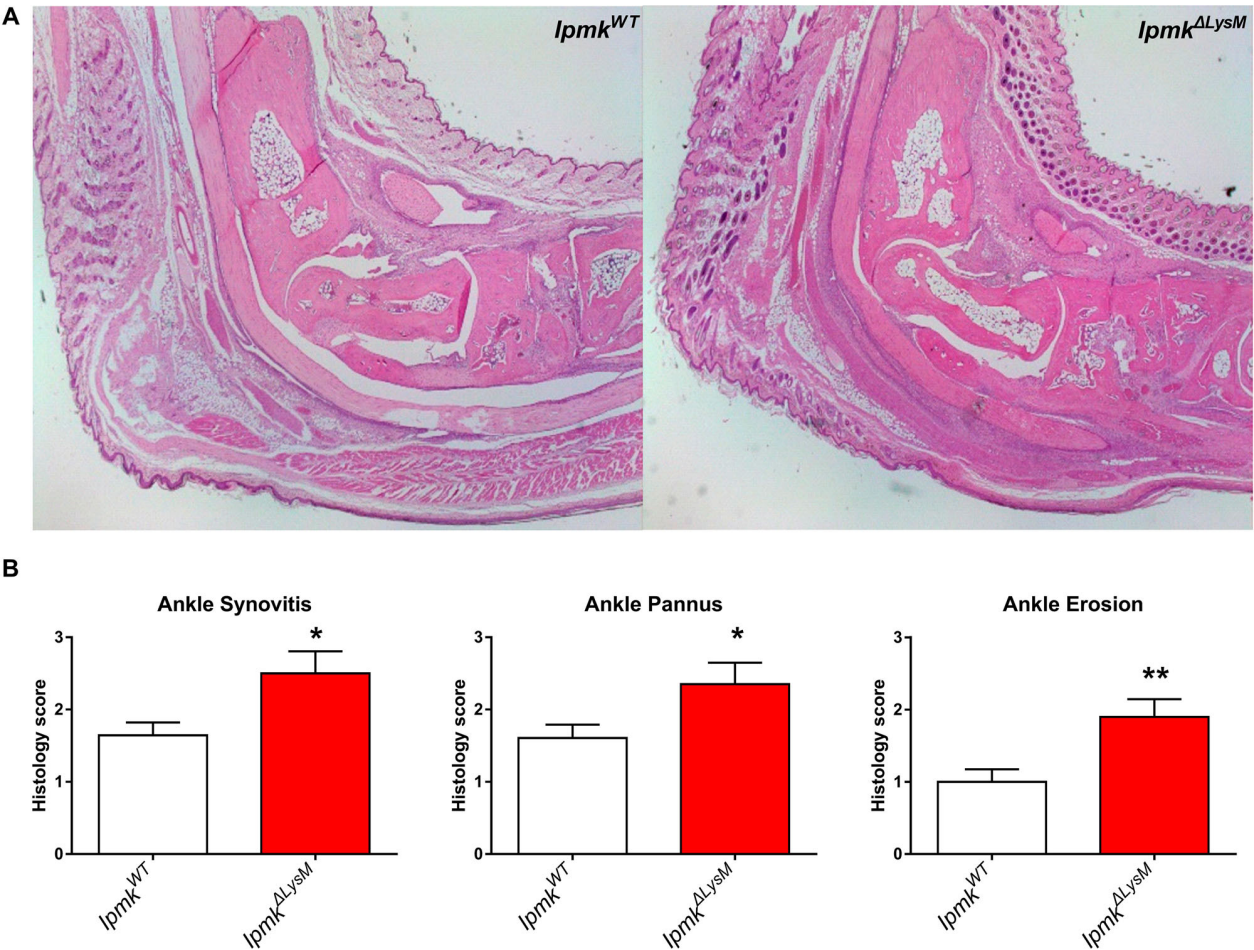
Myeloid IPMK reduces synovitis, pannus formation, and joint erosion. (A) Representative images of H&E staining of ankle joints from *Ipmk^ΔLysM^* or *Ipmk^WT^* mice in K/BxN serum-transfer arthritis (original magnification X20). (B) Histologic scores of synovitis, pannus formation, and bone erosion of ankle joints. Data are shown as mean ± SEM. *p < 0.05, **p < 0.01.

To investigate the role of myeloid IPMK in inflammatory arthritis, we induced K/BxN serum-transfer arthritis in wild-type (*Ipmk^WT^*) or myeloid cell-specific IPMK KO mice (LysM-Cre^+^*Ipmk^fl/fl^* designated as *Ipmk^ΔLysM^*) by injecting serum from K/BxN mice. Although both *Ipmk^WT^* and *Ipmk^ΔLysM^* mice developed arthritis, *Ipmk^ΔLysM^* mice exhibited more severe arthritis than wild-type (Figure 1). In particular, while *Ipmk^WT^* mice showed normal resolution of inflammation after day 14, deletion of myeloid IMPK delayed the resolution of inflammation, which suggests that myeloid IPMK may be related to the resolution phase of inflammation. Histologic analysis revealed that *Ipmk^ΔLysM^* mice showed greater synovitis, pannus formation, and erosion in the ankle joints compared with *Ipmk^WT^* mice (Figure 2). These findings demonstrate that myeloid IPMK is involved in inhibiting the severity of inflammatory arthritis by promoting the resolution of inflammation.

### Deletion of myeloid IPMK does not affect Fc receptor signaling

**Figure 3.**
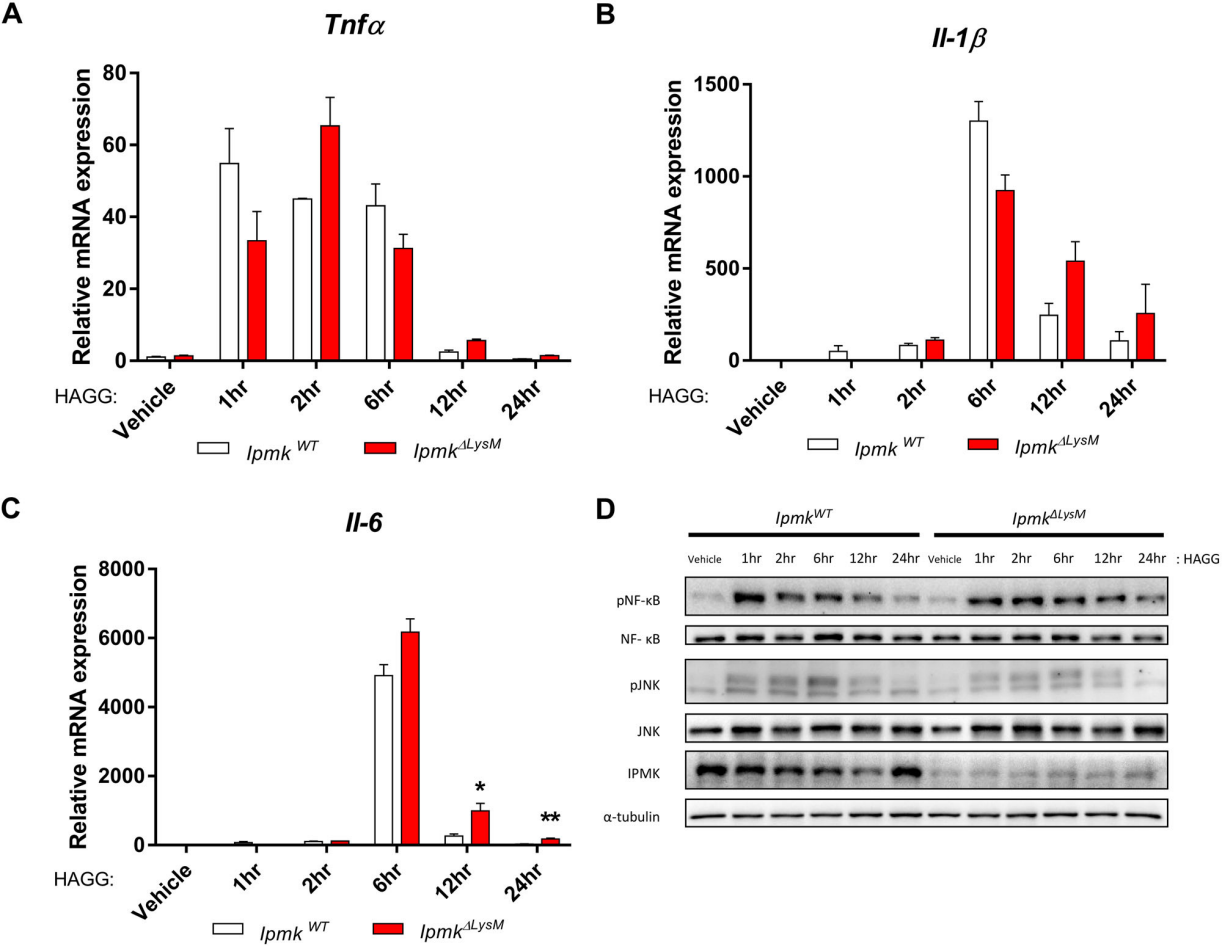
Macrophage IPMK does not impair FcR signaling. mRNA expression of the proinflammatory cytokines *Tnf-α* (A), *Il-1β* (B), and *Il-6* (C) was quantified by RT-qPCR in *Ipmk^ΔLysM^* and *Ipmk^WT^* BMDMs treated with HAGG (25 μg/ml) on designated time point. Data are shown as means ± SEM (*n* = 3). *p < 0.05, **p < 0.01. (D) Phosphorylation of NF-κB and JNK was analyzed from *Ipmk^ΔLysM^* and *Ipmk^WT^* BMDM lysates treated with HAGG (25 μg/ml) by immunoblotting. Images shown are representative of experiments performed twice.

Injection of K/BxN serum containing high concentrations of glucose-6-phosphate isomerase-specific IgG antibodies primarily leads to inflammatory responses via FcR activation (Sánchez-Mejorada and Rosales 1998). To determine whether myeloid IPMK contributes to Fc receptor signaling, which is a major effector of immune responses in rheumatoid arthritis, bone-marrow-derived macrophages (BMDMs) isolated from *Ipmk^ΔLysM^* and *Ipmk^WT^* littermates were treated with a heat-aggregated gamma globulin (HAGG) immune complex, after which the mRNA levels of pro-inflammatory cytokines such as *Tnf-α*, *Il-1β*, and *Il-6* were analyzed. Interestingly, these pro-inflammatory cytokines were similarly induced in both *Ipmk^ΔLysM^* and *Ipmk^WT^* BMDMs in response to HAGG treatment (Figure 3(A–C)). Further, no differences were observed between the phosphorylation levels of NF-κB (nuclear factor kappa-light-chain-enhancer of activated B cells) and JNK (c-Jun N-terminal kinase) in the HAGG-treated *Ipmk^ΔLysM^* and *Ipmk^WT^* BMDMs (Figure 3(D)). Collectively, our findings demonstrate that FcR signaling was largely unaffected by IPMK loss in macrophages.

### Abolishment of myeloid IPMK does not affect osteoclast differentiation

**Figure 4.**
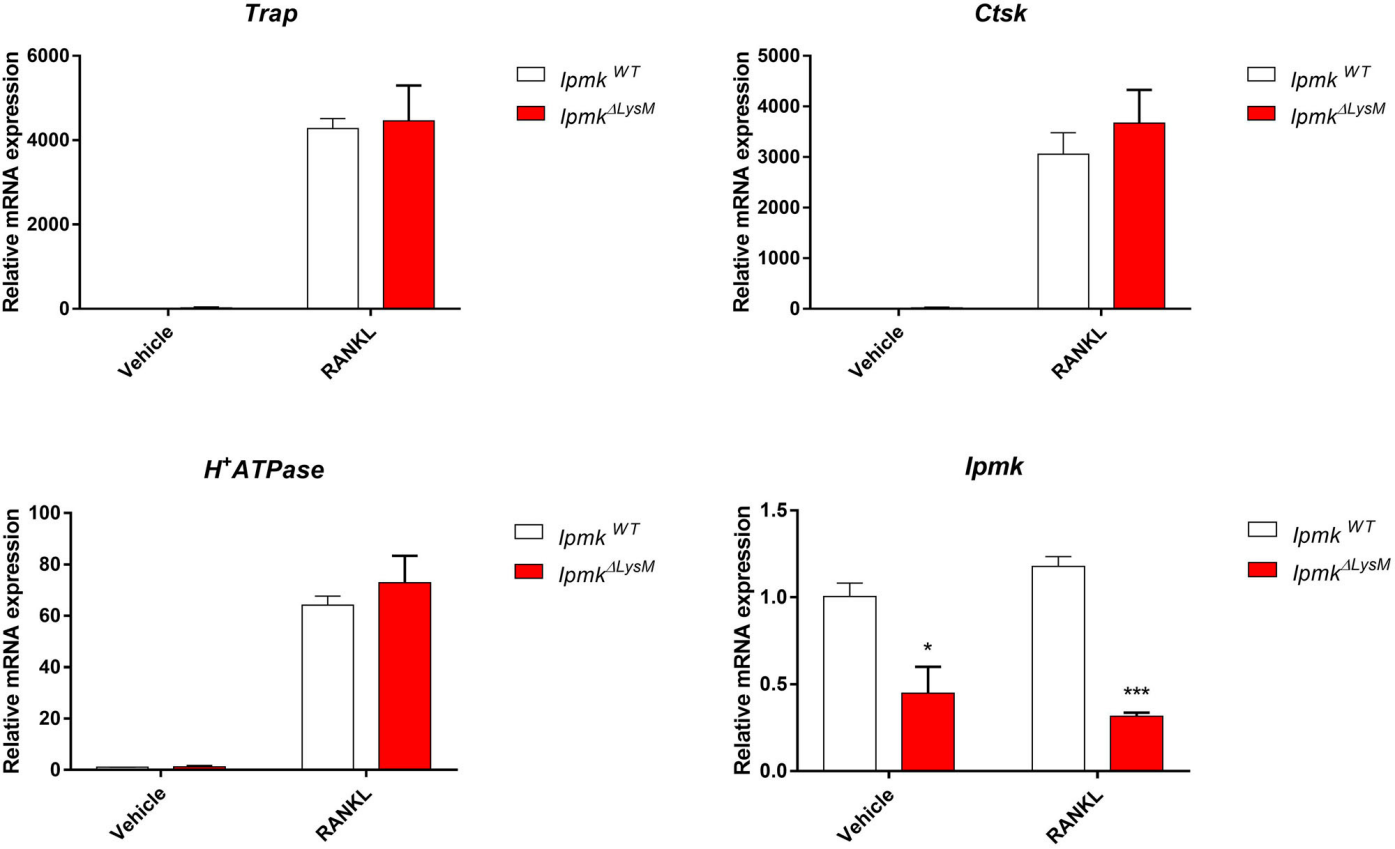
Myeloid IPMK deletion does not alter osteoclast differentiation. mRNA expression of the markers for osteoclast differentiation *Trap*, *Ctsk*, *H^+^-ATPase* was quantified by RT-qPCR in *Ipmk^ΔLysM^* and *Ipmk^WT^* BMDMs after 5 days treatment with M-CSF (50 ng/ml) and RANKL (100 ng/ml). Data are shown as means ± SEM (*n* = 3). *p < 0.05, **p < 0.01, ***p < 0.001.

Osteoclasts differentiated from monocyte lineages are known to contribute to bone erosion and degeneration, which are major symptoms of arthritis (Schett and Gravallese 2012). To investigate whether IPMK is involved in osteoclast differentiation, isolated BMDMs were differentiated for 5 days in the presence of osteogenic cytokine RANKL (Receptor activator of nuclear factor kappa-B ligand). However, no differences were observed between the levels of osteoclast differentiation markers such as tartrate-resistant acid phosphatase (*Trap*), cathepsin K (*Ctsk*), and H^+^-ATPase in *Ipmk^ΔLysM^* and *Ipmk^WT^* BMDMs (Figure 4). Therefore, these results suggest that myeloid deletion of IPMK does not affect RANKL-stimulated osteoclastogenesis.

### Absence of myeloid IPMK does not alter resolvin signaling

**Figure 5.**
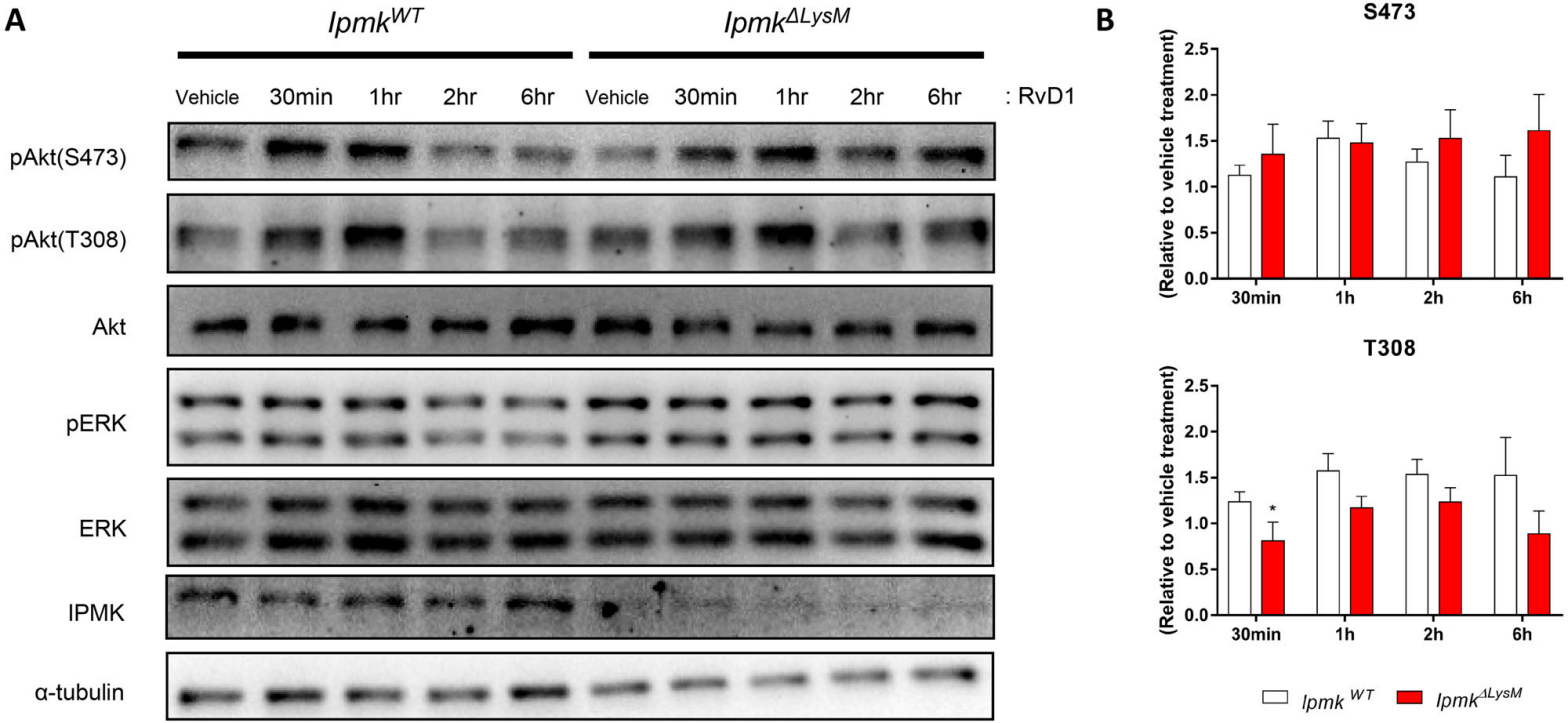
Macrophage IPMK is dispensable for resolvin D1 signaling. (A) Phosphorylation of Akt and ERK was analyzed by immunoblotting. Lysates of *Ipmk^ΔLysM^* and *Ipmk^WT^* BMDMs were prepared by resolvin D1 (50 nM) treatment for designated timepoint. (B) All blots are representative of four independent experiments. Densitometric quantitation results were normalized to ethanol-treated controls. Data are shown as means ± SEM (*n* = 4). *p < 0.05.

Resolvins, pro-resolving lipid mediators, are derived from polyunsaturated fatty acids, exert pro-resolving activities during the resolution phase of inflammation (Norling and Perretti 2013; Serhan and Levy 2018). To examine whether myeloid IPMK mediates inflammatory resolution through resolvin signaling pathways, we treated *Ipmk^ΔLysM^* or *Ipmk^WT^* BMDMs with resolvin D1 (RvD1), one of major resolvins synthesized from omega-3 fatty acid docosahexaenoic acid. As measured by phosphorylation levels of ERK and Akt, RvD1-treated *Ipmk^ΔLysM^* BMDMs did not show apparent downstream signaling changes, compared with *Ipmk^WT^* cells (Figure 5(A, B)), suggesting that IPMK is dispensable for RvD1 signaling activation in macrophages.

## Discussion

Rheumatoid arthritis, one of the most prevalent autoimmune diseases, affects nearly 1% of the world’s population (McInnes and Schett 2011). Nevertheless, although the pro- and anti-inflammatory roles of myeloid cells such as macrophages have been functionally linked to RA pathogenesis (Siouti and Andreakos 2019), the key molecular mechanisms that drive the progression of this disease have not been elucidated. Therefore, our study sought to characterize the physiological role of myeloid cell *Ipmk* expression in RA control via targeted deletion of *Ipmk* using the LysM-Cre/LoxP system. The K/BxN serum-transfer model was chosen because it mimics the effector phase of RA, which is primarily associated with the activities of myeloid cells (e.g. macrophages, neutrophils) (Christensen et al. 2016). Our results show that IPMK in lysozyme M-expressing cells suppresses inflammatory responses, as *Ipmk^ΔLysM^* mice exhibited more severe arthritic phenotypes such as clinical scores and paw thickness, in addition to histological indicators of disease progression such as synovitis, pannus formation, and bone erosion. Interestingly, IPMK deletion in myeloid cells delays the resolution of K/BxN serum-transfer induced arthritis without altering the initiation or developmental phase.

To identify the potential mechanisms by which myeloid IPMK could impact the resolution of RA inflammation, we analyzed key features of IPMK-deficient macrophages in response to various physiological upstream stimuli such as heat-aggregated gamma globulin and osteoclastogenic RANKL. Loss of IPMK in BMDMs did not influence normal responses in FcR signaling activation and osteoclast differentiation. We further tested whether macrophage IPMK could be involved in pro-resolving RvD1 signaling and observed that RvD1-stimulated IPMK KO BMDMs exhibited proper activation of Akt and ERK, which was comparable to that of wild-type cells. Collectively, these findings suggest that IPMK does not play a critical role in mediating major macrophage activities linked to RA-associated inflammatory events, indicating that myeloid IPMK may promote RA resolution through other pathways.

Resolution of inflammation is a complex process that takes place after acute immune responses. Unregulated resolution of RA thus leads to severe inflammation in the joint tissues with significant levels of chronicity (Fullerton and Gilroy 2016; Schett and Neurath 2018). Our findings indicated that IPMKs are not necessary for FcR activation, osteoclastogenesis, and RvD1 signaling; however, future studies should further characterize the role of IPMK-mediated macrophage activities in other mechanisms of RA progression. During RA resolution, immune cells such as neutrophils infiltrate the inflamed joint, thus initiating apoptosis and necrosis. Macrophages then engulf apoptotic cells to clear up the tissues through a process called efferocytosis. Upon the transformation of macrophages from classically activated to alternatively activated cells, immune cells return from the joint to the vasculature or lymph vessels, thus initiating the resolution process and normal tissue homeostasis (Serhan and Savill 2005). Therefore, additional studies are needed to explore the role of macrophage IPMK in efferocytosis and related macrophage polarization. In addition to macrophages, the activities of IPMK in other myeloid cells such as neutrophils and dendritic cells warrant further investigation. It will be also important to dissect the contribution of IPMK-dependent IP metabolites including IP5 as well as versatile inositol pyrophosphate (Park et al. 2018; Lee et al. 2020).

In conclusion, our study demonstrated that myeloid IPMK promotes the resolution of arthritis in a K/BxN serum transfer arthritis model. Yokoyama et al. previously reported that a single-nucleotide polymorphism (SNP) in IPMK, which is associated with decreased expression of IPMK, was detected in samples from tissues of Alzheimer’s disease and immune-mediated diseases including RA, suggesting that IPMK is a risk factor for chronic inflammatory diseases (Yokoyama et al. 2016). Furthermore, a recent study reported that the IPMK expression levels in an adjuvant-induced arthritis rat model were lower after exercise-induced physical stress (González-Chávez et al. 2019). Taken together, the above-described evidence and our *in vivo* findings indicate that myeloid IPMK promotes RA resolution and is therefore a key molecular target with important clinical implications for RA treatment.

## Materials and methods

### Animals

KRN TCR transgenic mice were gifts from Diane Mathis (Harvard Medical School). To obtain arthritogenic serum, K/BxN mice were generated by crossing KRN TCR transgenic mice with NOD mice. Myeloid-specific IPMK knockout mice or their littermate control mice were bred in KAIST. All animal experiments were performed after institutional animal care and use committee approvals from Pusan National University and KAIST (IACUC #PNU-2020-2686, KA2018-52)

### K/BxN serum-transfer arthritis

The arthritogenic K/BxN sera were collected from K/BxN mice (8∼14 weeks old), and pooled and frozen at −70°C until use. Male myeloid-specific IPMK knockout mice or their littermate control mice (10∼12 weeks old) were injected intraperitoneally with 125 μl of K/BxN serum on days 0 and 2. To determine arthritis severity, each paw was assigned a clinical score of 0∼4, with a maximum score of 16 per mouse. Arthritis severity was evaluated as previously described (Song et al. 2011). Each limb was assigned a clinical score of 0 to 4, with a maximum score of 16 per mouse. Hind paw thickness was measured with a caliper on alternate days. At the termination of the experiment, hindlimbs from mice were fixed and decalcified for histological analysis.

### Histological assessment of arthritis

Sections of ankle tissues were stained with hematoxylin and eosin. Histological assessment of arthritis was scored by three investigators who were blinded to the experimental groups, as previously described (Paniagua et al. 2010).

### Statistical analysis

Statistical analysis was analyzed by using the Graphpad Prism software. Data are expressed as mean ± SEM. Analyses of differences were performed using unpaired t-test or one-way ANOVA. *p* values less than 0.05 were considered statistically significant.

### Bone marrow-derived macrophage (BMDM) isolation and resolvin D1 treatment

Bone marrow was isolated from femurs and tibias of 6–8 weeks old mouse and differentiated into BMDMs for 6 days in RPMI 1640 supplemented with 10% FBS, 1 mM sodium pyruvate, 2 mM L-glutamine, 100 mg/ml penicillin/streptomycin and 30 ng/ml recombinant M-CSF (R&D systems) on non-culture treated petri dishes. Adherent BMDMs were detached on day 6 and plated in multi well plates, and used for analysis on the day 7. Resolvin D1 (Cayman chemical) was treated on BMDM with 50 nM.

### Preparation of heat-aggregated gamma globulin (HAGG)

To generate HAGG which mimics immune complexes, IgG from mouse serum (Sigma Aldrich) at 5 mg/ml was incubated at 63°C for 30 min and placed on ice for 30 min. Then, IgG was centrifuged at 4°C, 10,000× *g* for 10 min. Supernatant was used for BMDM activation.

### RNA isolation and RT-qPCR

Total RNA was isolated from cells using the TRI Reagent (Molecular Research Center) according to the manufacturer’s protocol. Complementary DNA was synthesized from 0.35 to 2 μg of total RNA using reverse transcriptase (Enzynomics). RT-qPCR analyses were performed using SYBR Green Master Mix (Toyobo) (Lee et al. 2020) and the Step One Plus Real-Time PCR System (Applied Bio- systems). Expression levels of genes of interest were analyzed using the Ct method with 18s ribosomal RNA as the internal housekeeping control. Primer sequences for quantitative PCR are as follows: *18s*, forward 5-CGCTTCCTTACCTGGTTGAT-3, reverse: 5-GAGCGACCAAAGGAACCATA-3. *Ipmk*, forward: 5′-CCAAAATATTATGGCATCTG-3′; reverse: 5′-TATCTTTACATCCATTATAC-3′. *Tnf-α*, forward 5′-CACAAGATGCTGGGACAGTGA-3′, reverse: 5′-GAGGCTCCAGTGAATTCGGA-3′. *Il-1β*, forward: 5′-GCCTCGTGCTGTCGGACC-3′, reverse: 5′-TGTCGTTGCTTGGTTCTCCTTG-3′. *Il-6*, forward: 5′-ATGAACAACGATGATGCACTT-3′, reverse: 5′-TATCCAGTTTGGTAGCATCCAT-3′. *Trap*, forward: 5′-GCGACCATTGTTAGCCACATACG-3′, reverse: 5′-CGTTGATGTCGCACAGAGGGAT-3′. *Ctsk*, forward: 5′-AGCAGAACGGAGGCATTGACTC-3′, reverse: 5′-TTTAGCTGCCTTTGCCGTGGC-3′. *H^+^-ATPase*, forward: 5′-ACGGTGATGTCACAGCAGACGT-3′, reverse: 5′-CCTCTGGATAGAGCCTGCCGCA-3′.

### Immunoblot analysis

For immunoblot analysis, cells were washed twice with PBS and lysed in lysis buffer (1% NP-40, 120 mM NaCl, 40 mM Tris-HCl (pH7.4), 1.5 mM sodium orthovanadate, 50 mM sodium fluoride, 10 mM sodium pyrophosphate, and protease inhibitor cocktail (Roche)), as described in (Cai et al. 2020; Lee et al. 2020). Primary antibodies against phospho-Akt (9272), Akt (4056), phospho-ERK (9101), ERK (4696) (Cell Signaling), α-tubulin (T5169; Sigma), and horseradish peroxidase-conjugated secondary antibodies were used. IPMK antibody was generated with a peptide targeting mouse IPMK amino acids 295-311 (SKAYSRHRKLYAKKHQS) from Covance.

### Osteoclast differentiation

BMDM was cultured with same protocol as described above except using α-MEM instead of using RPMI 1640, supplemented with 10% FBS, 100 mg/ml penicillin/streptomycin and 30 ng/ml recombinant M-CSF (R&D systems). Then, BMDM was plated to 6-well plate with α-MEM including 50 ng/ml M-CSF and 100 ng/ml RANKL (R&D systems) for 5 days (Zhang et al. 2020).
